# 76-year-old gentlemen with primary cardiac lymphoma presenting as acute coronary syndrome and atrioventricular block

**DOI:** 10.1259/bjrcr.20150466

**Published:** 2016-07-28

**Authors:** Baskar Sekar, Gagan Swami, Amin Ibrahim, Azad Hanna, Mark N Payne, James RC Seale, Abdul Azzu

**Affiliations:** ^1^Cardiology Department, Ysbyty Gwynedd Hospital, Betsi Cadwaladr University Health Board, Bangor, UK; ^2^Haemotology Department, Ysbyty Gwynedd Hospital, Betsi Cadwaladr University Health Board, Bangor, UK

## Abstract

We report the case of an immunocompetent patient who presented with symptoms suggestive of acute coronary syndrome and was found to be in complete heart block. He re-presented within 2 months with worsening breathlessness and investigations confirmed infiltrative cardiac disease. We describe here an uncommon presentation of primary cardiac lymphoma as acute coronary syndrome and atrioventricular block.

## Case presentation

A 76-year-old immunocompetent male presented to our institution with a 1-week history of increasing breathlessness and intermittent chest tightness. He was an ex-smoker and his only significant past medical history was deep vein thrombosis and renal stones. On admission, he was found to be bradycardic with a heart rate of 47 beats per minute; clinical examination was otherwise unremarkable. His blood test showed an elevated cardiac troponin-T level of 224 ng ml^−1^ (reference range <0.01 ng ml^–1^) and electrocardiogram demonstrated complete heart block with narrow QRS complexes. He was diagnosed and treated as acute coronary syndrome. His chest X-ray was unremarkable and a repeat electrocardiogram the following day demonstrated second-degree atrioventricular block (Mobitz Type 2), but he remained haemodynamically stable. Transthoracic echocardiogram revealed mild hypertrophy of the ventricles with hypokinesis in the lateral wall but preserved left ventricular systolic function. He subsequently underwent successful percutaneous intervention with two drug-eluting stents to a significant stenosis in the circumflex artery. He remained in second-degree atrioventricular block 5 days later, hence a dual-chamber pacemaker was implanted and the patient was discharged home in stable condition.

**Figure 1.**
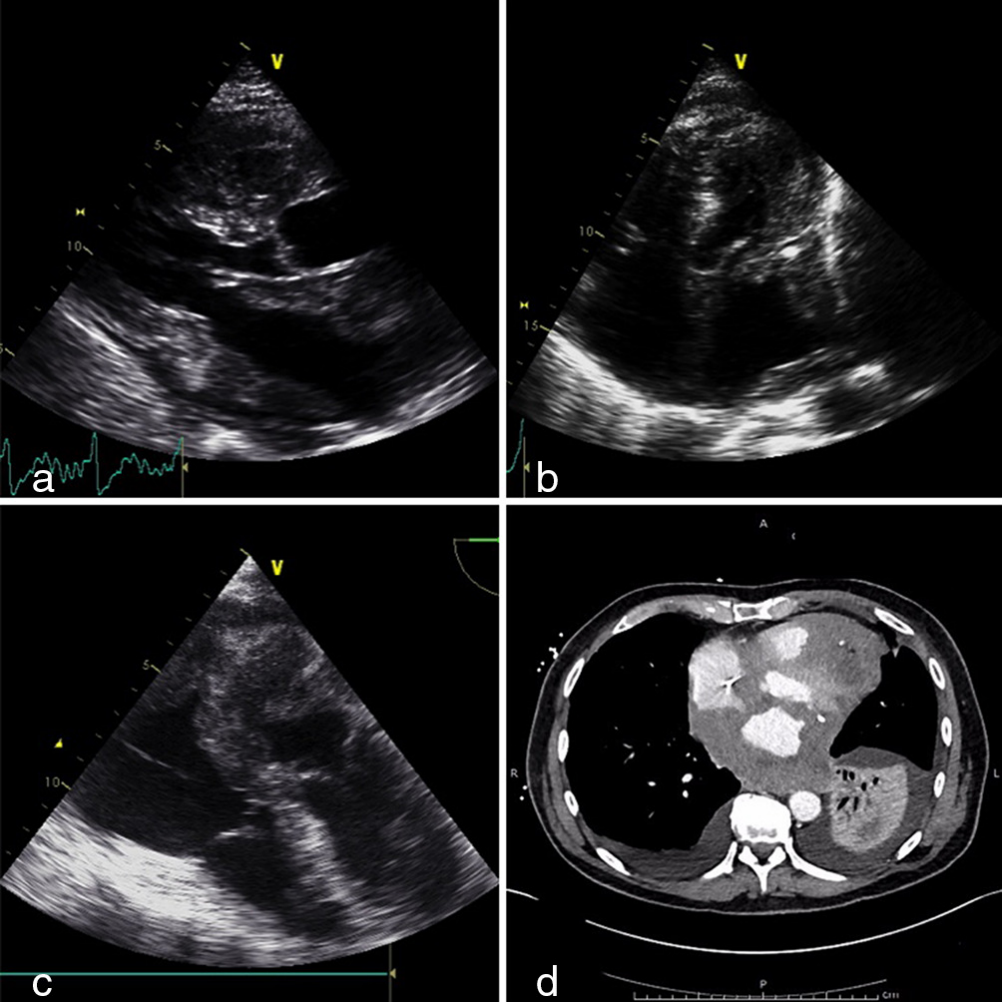
(a) Transthoracic echocardiogram (parasternal long axis view) showing marked infiltration of the left atrium and both the ventricles by diffuse large B-cell lymphoma. (b) Transthoracic echocardiogram (apical four-chamber view) showing marked infiltration of the left atrium and both the ventricles by the diffuse large B-cell lymphoma. (c) Transoesophageal echocardiogram (three-chamber view) showing extensive infiltration of the left atrium and the ventricles by diffuse large B-cell lymphoma. (d) CT imaging of the thorax showing extensive lymphoma infiltration of the heart.

**Figure 2.**
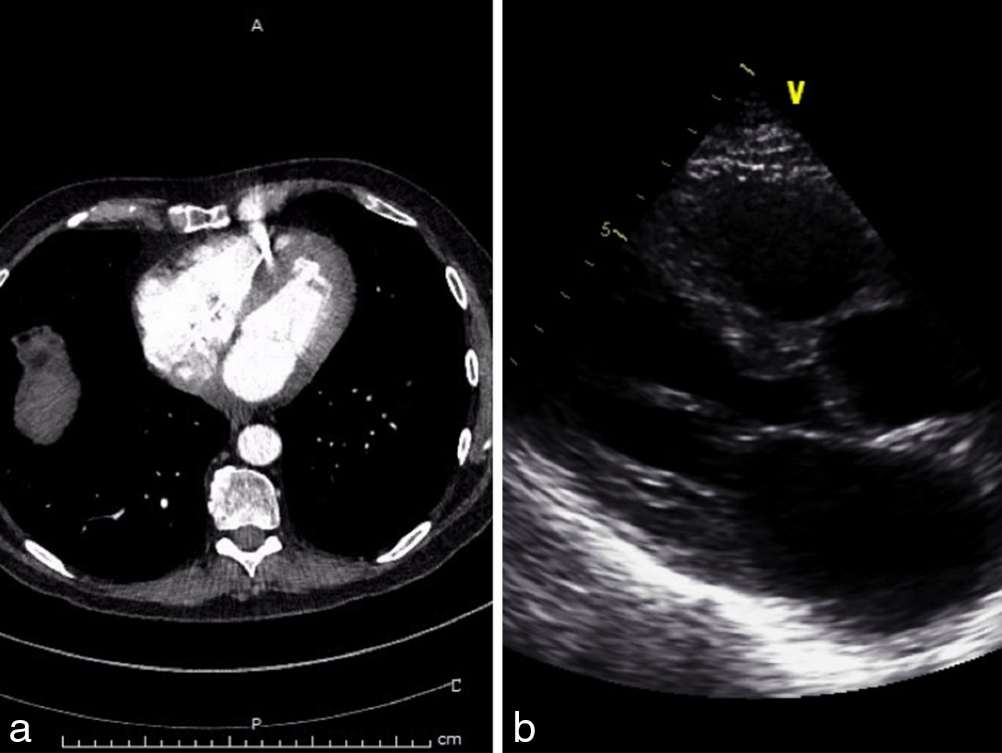
(a) CT of the thorax showing significant improvement in the appearance of the lymphoma following chemotherapy. (b) Transthoracic echocardiogram (parasternal long axis view) showing significant improvement in the appearance of cardiac chambers post chemotherapy.

**Figure 3.**
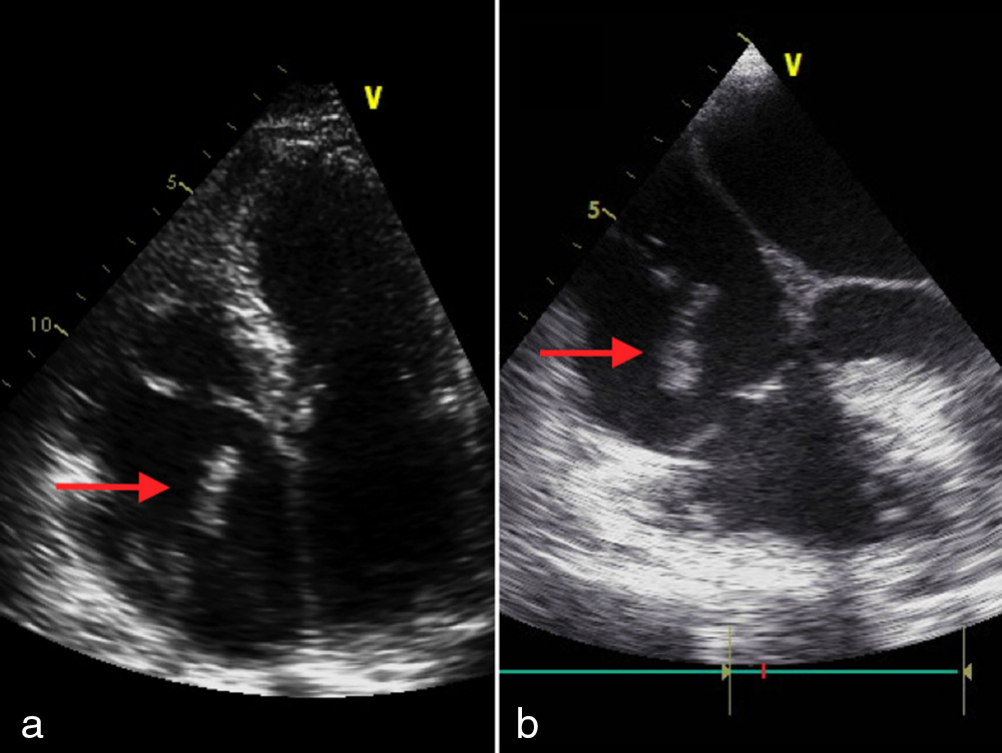
(a) Transthoracic echocardiogram (apical four-chamber view) showing a highly mobile thrombus in the right atrium attached to the pacing lead. (b) Transoesophageal echocardiogram (bicaval view) showing a highly mobile thrombus in the right atrium attached to the pacing lead.

**Figure 4.**
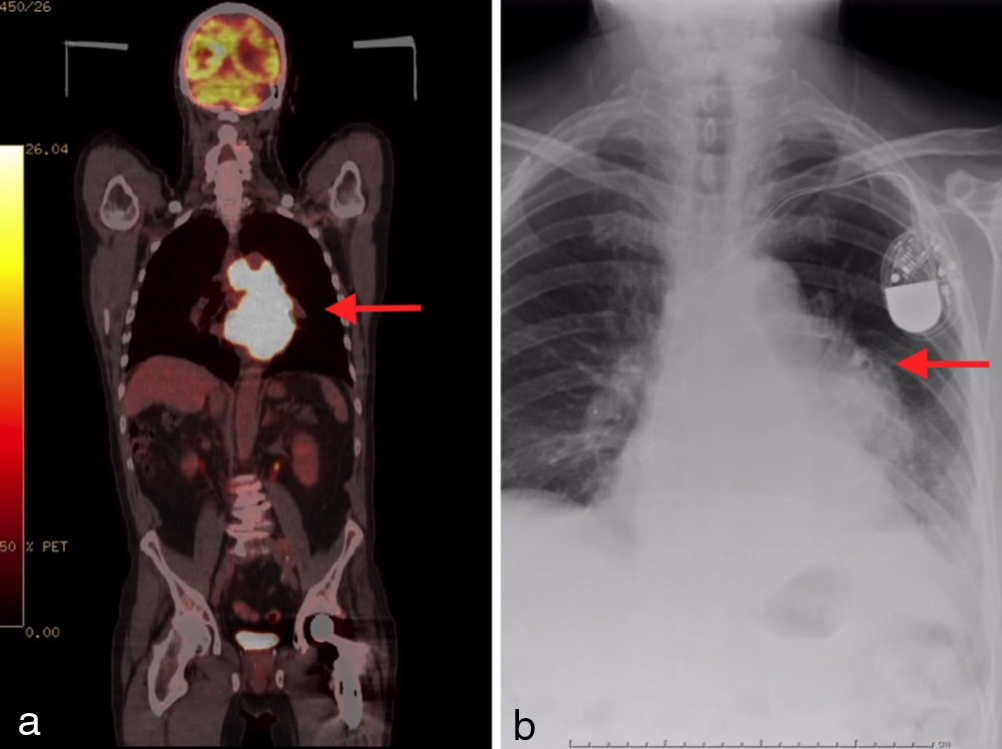
(a) Positron emission tomography showing an infiltrative mediastinal mass with very high intensity fludeoxyglucose uptake. (b) Chest X-ray showing a lobulated mass (lymphoma) at the left hilum associated with small left pleural effusion.

His general condition deteriorated soon after and he was readmitted within 2 months with worsening breathlessness and chest discomfort. Blood tests showed mild microcytic anaemia. Transthoracic echocardiogram and transoesophageal echocardiogram confirmed marked hypertrophy of both ventricles with granular speckled appearance suspicious of infiltrative disease. In addition, the left atrium was lined with an echogenic speckled mass encroaching on the the mitral valve leaflets (Figure 1a–c; Supplementary Video A). He denied any B symptoms. Myeloma screening was negative and rectal biopsy excluded amyloidosis. CT scan of his thorax confirmed bilateral pleural effusion and an extensive abnormal soft tissue infiltrating the pericardium, much of the myocardium and the inferior portion of the left atrium, extending up to the aortic arch into the superior mediastinum (Figure 1d). Pleural fluid cytology was inconclusive. The patient was commenced on i.v. dexamethasone and rasburicase for suspected lymphoma, with significant improvement in his general condition and resolution of his heart failure symptoms. He subsequently underwent mediastinosopic biopsy of the mass, which confirmed it to be high-grade non-Hodgkin’s lymphoma of B-cell type, with immunocytochemical stain positive for CD45 and CD20. He was commenced on rituximab, gemcitabine, cyclophosphamide, vincristine and prednisolone (R-GCVP), which is the preferred regimen for diffuse large B-cell lymphoma with cardiac involvement. He responded very well to the first two cycles of chemotherapy, achieving remission echocardiographically and radiologically (Figure 2a,b; Supplementary Video B). However, he could not tolerate intensification or extension of treatment beyond six courses. He had multiple hospital admissions during the course owing to neutropenic sepsis requiring antibiotics and intermittent cessation of chemotherapy. In addition, he also developed deep vein thrombosis in his left leg and thrombus attached to the pacing lead that required anticoagulation (Figure 3a,b; Supplementary Video C). A month after completing the chemotherapy, he re-presented with increasing breathlessness, low backache and chest discomfort and a positron emission tomographic scan confirmed a metabolically active mediastinal mass, which was consistent with recurrence (Figure 4a). Although the tumour had originally responded quite well to chemotherapy, it had recurred very quickly and at a very rapid rate. He could not tolerate more intensive chemotherapy, hence palliative oral chemotherapy (DECC regimen) consisting of dexamethasone, etoposide, chlorambucil and lomustine was initiated. Despite initial stabilization, he continued to deteriorate, and a repeat chest X-ray confirmed clear evidence of lymphoma progression (Figure 4b). Chemotherapy was discontinued after discussion with the patient and family and he passed away peacefully at home a week later.

## Discussion

Primary cardiac lymphoma is exceptionally rare, constituting 1% of all primary cardiac tumours.^1^ Of these, diffuse large B-cell lymphoma is the most common, representing a majority of the cases.^2^ Primary cardiac lymphomas occur commonly in immunosuppressed conditions such as human immunodeficiency virus infection and post-cardiac transplantation, and frequently affect the right heart.^3^ In contradistinction, the left heart was predominantly affected in our immunocompetent patient. Owing to their heterogeneous presentations, such as heart failure, embolic events and arrhythmias, they often pose greater difficulties in making the diagnosis. Although imaging modalities such as echocardiography, CT scan and MRI are helpful in the initial diagnosis, histopathological analysis of the biopsied specimen is required for a definitive diagnosis. Chemotherapy remains the mainstay of treatment with R-GCVP and R-CHOP (rituximab, hydroxydaunorubicin, vincristine sulfate and prednisone) being the most commonly used regimens. Our patient was treated with R-GCVP regime owing to preferable cardiotoxic profile compared with R-CHOP.^4^ Prognosis remains poor and these patients should be closely monitored owing to complications secondary to tumour *per se,* chemotherapy and also risk of early relapse, as demonstrated in this case.

## Learning points

1. Primary cardiac lymphoma is an exceptionally rare cardiac tumour that commonly affects the right heart in an immunosuppressed patient. Involvement of the left heart in an immunocompetent individual is very unusual.
2. Imaging modalities such as echocardiography, CT scan and MRI are helpful in the initial diagnosis. However, histopathological analysis of the biopsied specimen is often required for making a definitive diagnosis.
3. Chemotherapy remains the mainstay of treatment and these patients should be kept under surveillance owing to complications from chemotherapy and the risk of early relapse.

## Consent

Informed consent to publish this case (including images and data) was obtained and is held on record.

## References

[1] Patel J, Melly L, Sheppard MN Primary cardiac lymphoma: B- and T-cell cases at a specialist UK centre. Ann Oncol 2010; 21: 1041–5. 19846467 10.1093/annonc/mdp424

[2] Reynen K Frequency of primary tumors of the heart. Am J Cardiol 1996; 77: 107. 8540447 10.1016/s0002-9149(97)89149-7

[3] Sheppard MN, Angelini A, Raad M, Savelieva I Tumours of the heart In: Camm AJ, Luscher TF, Serruys PW, eds. The ESC textbook of cardiovascular medicine. London UK: Blackwell Publishing; 2006 535–52.

[4] Fields PA, Townsend W, Webb A, Counsell N, Pocock C, Smith P, et al De novo treatment of diffuse large B-cell lymphoma with rituximab, cyclophosphamide, vincristine, gemcitabine, and prednisolone in patients with cardiac comorbidity: a United Kingdom National Cancer Research Institute trial. J Clin Oncol 2014; 32: 282–7. 24220559 10.1200/JCO.2013.49.7586

